# Supplementary material for: MAF: An algorithm based on multi-agent characteristics for infrared and visible video fusion
Source: PLoS One. 2025 Mar 4;20(3):e0315266. doi: 10.1371/journal.pone.0315266 (PMC11878920; doi:10.1371/journal.pone.0315266)
Supplement: Attachment — Submitted filename: Response to Reviewers.docx [file pone.0315266.s001.docx]

**List of Responses**

**Dear Editors and Reviewers:**

Thank you for your letter and for the reviewers’ comments concerning our manuscript entitled "MAF: An Algorithm Based on Multi-Agent Characteristics for Infrared and Visible Video Fusion" (ID: PONE-D-24-28516). Those comments are all valuable and very helpful for revising and improving our paper, as well as providing important guidance for our research. We have carefully studied these comments and made the necessary corrections, which we hope meet with your approval. The revised portions are marked in red in the manuscript.

The main corrections in the paper and our responses to the reviewers' comments are as follows:

**Responses to the reviewers' comments:**

**Reviewer #1:**

1. The description of the multi-agent system and fusion agents lacks technical details. The authors should provide a more rigorous mathematical formulation of their proposed model.

Our actions：We have provided a more detailed description of the multi-agent fusion model and its decision-making process in the methods section. We have added mathematical definitions and notation for the fusion model, and established a clear mathematical model for the decision-making and interaction mechanisms of the fusion agents.

Finally, the fusion strategy is optimized through the interaction and collaboration among different agents to achieve global fusion optimization, as show in Eq (1).

$$MF=\{\text{ }AF,P,S,U,C,K\}$$

Where. ***AF*** denotes Agent Function, referring to the function set of the agent, encompassing the specific tasks that each agent performs during the fusion process. ***P*** represents Perception Function, which is used to extract the difference features in video frames and identify the main difference feature of the current frame. ***S*** represents Strategy Space, which includes the combination of fusion algorithms, fusion rules, and fusion parameters, defining all possible fusion strategies. ***U*** represents Utility Function, which is used to evaluate the effectiveness of different fusion strategies and select the optimal strategy. ***C*** represents Collaboration Mechanism, which defines how multiple agents collaborate to optimize the global fusion task. K represents the knowledge base, which is used to store the optimal fusion strategies accumulated by the agent during the fusion process for use in future similar tasks. The main process is illustrated in Fig. 1.

The perception function is defined as show in Eq(4) :

$$P(i)=\arg\max_{m\in M} \frac{D_{i,m}-min(D_{1,m},D_{2,m},\ldots,D_{n,m})}{max(D_{1,m},D_{2,m},\ldots,D_{n,m})-min(D_{1,m},D_{2,m},\ldots,D_{n,m})}$$

The multi-agent fusion model utilizes the perception function $P\left(i\right)$ to identify the main difference feature type from the input video sequence at frame *i*. Specifically, $P\left(i\right)$outputs the main difference feature type by analyzing the difference characteristics between the infrared and visible light frames. This output $P\left(i\right)$ serves as the basis for the fusion agent to subsequently select the optimal fusion strategy, based on the identified primary characteristic.

The complete strategy combination is shown in Eq(5):

$$S=\{(a,b,\theta)\text{∣}a\in A,r\in R,\theta\in\text{Θ}\}$$

Here, the utility function represents the fusion effect on the i-th frame, for the main difference feature m, using fusion strategy S, the larger the value, the higher the fusion effectiveness. as shown in Eq(7).

$$U_{i,m}(a,r,\theta)=w_{i,m}^{\text{I}}\times SIM\left(D_{i,m},D_{i,m}^{\text{I}}\right)\text{+}w_{i,m}^{\text{V}}\times SIM\left(D_{i,m},D_{i,m}^{\text{V}}\right)$$

Where.

$$\left\{\begin{matrix} SIM\left(D_{i,m},D_{i,m}^{\text{I}}\right)=\frac{D_{i,m},D_{i,m}^{\text{I}}}{\sqrt{\left(D_{i,m}\right)^{2}+\left(D_{i,m}^{\text{I}}\right)^{2}}} \\ SIM\left(D_{i,m},D_{i,m}^{\text{V}}\right)=\frac{D_{i,m},D_{i,m}^{\text{V}}}{\sqrt{\left(D_{i,m}^{k}\right)^{2}+\left(D_{i,m}^{\text{V}}\right)^{2}}} \end{matrix}\right.$$

$$\left\{\begin{matrix} \langle D_{i,m},D_{i,m}^{\text{I}}\rangle= D_{i,m}\cdot D_{i,m}^{\text{I}} \\ \langle D_{i,m},D_{i,m}^{\text{V}}\rangle= D_{i,m}\cdot D_{i,m}^{\text{V}} \end{matrix}\right.$$

$$\left\{\begin{matrix} w_{i,m}^{I}=\frac{D_{i,m}^{\text{I}}}{D_{i,m}^{\text{I}}+D_{i,m}^{\text{V}}} \\ w_{i,m}^{V}=\frac{D_{i,m}^{\text{V}}}{D_{i,m}^{\text{I}}+D_{i,m}^{\text{V}}} \end{matrix}\right.$$

$w_{i,m}^{I}$and $w_{i,m}^{V}$ denote the respective weights of differential features attributed to the infrared and visible images. $D_{i,m}$represents the magnitude of the primary differential feature in the fusion result of the i-th frame.

2. The structure of the paper should be improved for clarity. The methods section in particular jumps between concepts without a clear logical flow.

Our actions：We have reorganized the content of the methods section. In combination with the response to the first comment, we have ensured that each component of the fusion model is presented step by step in a logical sequence by providing a rigorous mathematical definition of the multi-agent system. Based on your suggestion, we have revised the discontinuous concepts in Section 2 and Section 3.

3. More details are needed on the datasets used.

Our actions：we have added detailed information about the dataset in the manuscript, making the content more comprehensive

The BEPMS dataset is commonly used for qualitative and quantitative comparisons in the field of image fusion. This dataset involves a scene of a soldier in camouflage walking through dense forest. In this experiment, our focus is on the first 200 frames of this dataset. OTCBVS contains 6 subcategories of datasets, including 17,089 infrared and visible light images from different scenes. In this paper, we use the second subcategory, "Ohio State University (OSU) Color-Thermal Database," with "Location 2" as the experimental group. The scene is located at a busy intersection on the Ohio State University campus, with 6 optical/thermal imaging sequences (3 per location), and the image size is 320 × 240 pixels. The INO dataset includes infrared and visible light videos captured under different weather conditions, featuring pedestrians, cars, and lawns in modern urban scenes, which can fully test the target detection capability of the algorithm.

4. The paper lacks some related fusion papers in the literature review, includes [Mitigating modality discrepancies for RGB-T semantic segmentation] [Superthermal: Matching thermal as visible through thermal feature exploration] [Matching as color images: Thermal image local feature detection and description], etc.

Our actions：we have revisited and supplemented the relevant literature, particularly the latest research on modality fusion and RGB-T fusion. In the revised literature review, we have highlighted the contributions of these studies in addressing modality discrepancies, thermal and visible image matching, as well as local feature detection and description, making our problem statement more convincing.

In recent years, scholars have increasingly acknowledged that mining and utilizing image feature information to guide image fusion is an effective approach to enhancing fusion quality and improving subsequent tasks. Lu et al[2]. introduced a triplet-based Siamese CNN for feature detection and extraction in thermal images. Subsequently, they presented SuperThermal[3], an end-to-end neural network designed for thermal feature learning. In our research, we propose a feature detection network inspired by the Faster-RCNN framework, integrating thermal enhancement and a multi-scale descriptor network to better capture feature representations in texture-less thermal images. Zhao et al[4]. developed a novel network for RGB-T semantic segmentation, effectively capturing contextual information. Nai et al[5]. proposed a dynamic feature fusion method based on spatiotemporal context, which deeply analyzes the characteristics of multiple visual features, leveraging the strengths of various features to address complex appearance changes and background clutter, thereby improving object tracking and detection performance.

Thank you very much for pointing out these key references; your feedback has been extremely helpful in improving our paper. We will try our best to reply to your comments. If the reply is not satisfactory, we hope you can give us another chance to modify it until you are satisfied. Because it is very important for us to publish this work on PLOS ONE.

**Reviewer #2:**

1. In the introduction section, elaborate in more detail on the limitations of infrared and visible light video fusion in existing methods, particularly the issue of dynamically adjusting fusion strategies. Clearly indicate the potential and advantages of multi-agent systems in solving this problem. Cite more recent literature on multi-agent systems and video fusion in the introduction to enhance the theoretical foundation of the paper. For example: FusionPID, FusionCPP, FusionJPSI, FusionIPCS, ICIF, etc.

Our actions：We have emphasized the limitations of existing infrared and visible light video fusion methods. Additionally, based on your suggestion, we have incorporated the latest literature on video fusion, demonstrating the connection and innovation of our approach compared to existing methods. Building on this, we have elaborated on the unique advantages of multi-agent systems in addressing this problem, particularly their distributed decision-making, real-time responsiveness, and collaborative optimization strategies in dynamic environments, highlighting their ability to intelligently fuse different data modalities in complex scenarios.

However, most fusion methods primarily depend on prior knowledge of the video, such as predetermined fusion algorithms or trained fusion models. These methods aim to identify efficient feature extraction techniques to extract source image features as comprehensively as possible, achieving satisfactory fusion results to a certain extent. In practical applications, the complexity and variability of video scenes mean that predetermined fusion models are unable to satisfy the fusion demands of continuously changing difference features. As a result, methods that rely on specific transformations or representations have become a bottleneck in improving fusion quality. Pre-selected fusion algorithms may lead to unsatisfactory results, and in severe cases, even cause fusion failure.

Dong et al[6-8]. proposed a fusion method that employs image feature information as a weight reference for the fusion strategy, allowing image feature information to be fed back into the image fusion process. They introduced a novel fusion framework based on cybernetics, called FusionPID[9], which utilizes a proportional-integral-derivative (PID) control system to fuse infrared and visible images. FusionPID takes advantage of the control system's feedback capabilities for various fusion tasks, ensuring the fused image retains both the thermal radiation from the infrared image and the texture from the visible image. Later, they presented a collaborative fusion method for infrared and visible images based on pulse-coupled neural networks (PCNN) and PID control systems, called FusionCPP[10]. The controller adaptively adjusts the fusion weight according to the differences, enabling precise fusion of image features into the new image. These methods offer valuable insights for the multi-agent video fusion discussed in this paper.

Research indicates that the functional architecture of agents highly aligns with the video fusion process.[11]. The deliberative agent[12], being the most intelligent type, can rigorously infer and reflect based on its understanding of the environment and its own intentions, making detailed action plans for collaboration and tasks. This aligns with the requirements of choosing optimal fusion strategies in video fusion. The self-learning agent updates its knowledge base in response to environmental changes[13], continually improving over time, consistent with the characteristics of storing fusion strategy knowledge bases during the fusion process. The reactive agent can respond quickly to external information[14], utilizing its rapid responsiveness to directly access fusion strategies from the existing knowledge base. Under a multi-scale fusion framework, the multi-agent fusion model achieves complex functions such as dynamic selection of fusion parameters, rules and algorithms through the collaboration of simple agents, while reducing system complexity and enhancing system robustness, reliability and flexibility.

In summary, agents can autonomously perceive changes in the external environment and respond accurately. Through collaboration with other agents, they formulate behavior strategies suitable for completing complex integrated tasks, providing a theoretical tool for efficiently selecting fusion strategies. Therefore, this paper addresses the issue of existing fusion models being unable to dynamically adjust fusion strategies based on video differences and ensuring full performance, by proposing an infrared and visible video fusion algorithm based on multi-agent characteristics. It constructs fusion agents with varying difference feature types, where agents internally infer and decide on the optimal fusion parameters, rules and algorithms. Through inter-agent collaboration, the optimal fusion structure is achieved, overcoming the problems of traditional models that cannot dynamically change fusion strategies.

2. Provide a detailed description of the various components and interaction mechanisms of a multi-agent system, including how to define the functions of the agent, how to perceive the environment, and how to make decisions.

Our actions：In Section 3 of the paper, we have provided rigorous mathematical definitions for the perception functions, utility functions, and decision-making in the fusion methods of the multi-agent fusion model. The entire Section 3 (Methods) discusses the functionality of each component of the multi-agent fusion model and the decision-making process.

3. Provide more detailed algorithm steps, including how to select appropriate fusion algorithms, rules, parameters, and structures based on differential features. Add pseudocode or flowcharts to help understand.

Our actions：We have reorganized the methods section, revising the illogical concepts and flowcharts. A more rigorous decision-making process has been provided, detailing the steps from the input of video sequences to the output of the fused images.

In this section, we first develop a multi-agent video fusion model MF. Then, we discuss the perception function and strategy space of the fusion model. Finally, we explore the process of selecting the optimal fusion strategy. The infrared and visible light video fusion algorithm we propose, based on the characteristics of multi-agent systems, analyzes the relationship between multi-agent characteristics and video fusion. According to the fusion requirements, a flexible and variable fusion model is constructed, allowing the fusion strategy to change according to difference feature variations. The multi-agent video fusion model invokes different fusion agents by detecting the main difference features in the video sequences to be fused. The fusion agent calculates the main differential features of the current video frame using the perception function and selects the optimal fusion strategy through the strategy space and utility function. Finally, the fusion strategy is optimized through the interaction and collaboration among different agents to achieve global fusion optimization, as show in Eq (1).

$$MF=\{\text{ }AF,P,S,U,C,K\}$$

Where. AF denotes Agent Function, referring to the function set of the agent, encompassing the specific tasks that each agent performs during the fusion process. P represents Perception Function, which is used to extract the difference features in video frames and identify the main difference feature of the current frame. S represents Strategy Space, which includes the combination of fusion algorithms, fusion rules, and fusion parameters, defining all possible fusion strategies. U represents Utility Function, which is used to evaluate the effectiveness of different fusion strategies and select the optimal strategy. C represents Collaboration Mechanism, which defines how multiple agents collaborate to optimize the global fusion task. K represents the knowledge base, which is used to store the optimal fusion strategies accumulated by the agent during the fusion process for use in future similar tasks. The main process is illustrated in Fig. 1.

Fig.1. Overall flowchart of Multi-agent fusion model

The critical factor in selecting the optimal fusion strategy is the perception function's ability to determine the number of main difference feature types present in the video frames to be fused. If the video frame contains only one main difference feature, a single fusion agent is sufficient to make step-by-step decisions on the fusion algorithm, rules, and parameters, selecting the fusion strategy based on the utility function values. However, when the video frame contains multiple main difference features, collaboration between fusion agents must be considered to achieve global optimization of the fusion effect.

4. Explain why these specific fusion algorithms and parameters were chosen, and discuss the importance of these choices in improving fusion performance. Meanwhile, explain how to evaluate the effectiveness of the model, including the selection of evaluation indicators and calculation methods.

Our actions：In Section 3.2, we analyzed the advantages of specific algorithms and parameters in the strategy space of the multi-agent fusion model. Additionally, in Section 4.3.1, we explained the evaluation metrics, their criteria for assessing fusion effectiveness, and the calculation methods.

The fusion results of each frame, based on the selected optimal fusion strategy, are discussed. Subjective evaluations are prone to influence from the evaluator's personal psychology and mental state, introducing a degree of subjectivity. Therefore, we selected 7 objective evaluation metrics to evaluate the fusion results of each method, and the fusion effect is directly proportional to the metric values.

The evaluation metrics include: the fusion volume function ($Q^{AB/F}$)[42], the weighted quality assessment metric ($Q_{w}$)[43], the universal image quality index ($Q_{0}$)[44], a structure similarity-based metric ($Q_{e}$)[45], mutual information ($MI$)[46], spatial frequency ($SF$)[47], and visual information fidelity ($VIFF$)[48].

$Q^{AB/F}$ is a function that represents the information from each source image in the fused image. The higher the $Q^{AB/F}$ value, the more information from each source image is retained. $Q^{AB/F}$ ranges from 0 to 1, with $Q^{AB/F}$ = 0 indicating total loss of input information and $Q^{AB/F}$ = 1 representing an ideal fusion result with no loss of input information. $Q_{0}$ measures the structural distortion between the fused image and the source images; the closer the value is to 1, the better the fusion performance. The $Q_{w}$ metric reflects the degree to which the fused image retains information from the original images, as well as their combined effect. The value ranges from 0 to 1, with higher values indicating better image quality and fuller retention of source image information. $Q_{e}$ is used to evaluate the edge-based fusion performance, with values ranging from 0 to 1. Higher similarity is indicated by values closer to 1. $MI$ measures the fusion results of infrared and visible light images, with the average value taken as the final output. With higher values indicating that more source image features are included in the fusion result. $SF$ is based on gradient distribution and effectively reveals image details and textures. $VIFF$ reflects the distortion in visual information between the fused image and the source images from the perspective of the human visual system. A larger value indicates better fusion quality.

5. Provide a detailed list of all parameter settings, hardware configurations, and software environments used during the experimental process, so that other researchers can reproduce the experimental results.

Our actions：In Section 4.3, we provided detailed descriptions of the algorithm parameters, hardware configurations, and software used in the experiments.

In order to verify the rationality and effectiveness of the proposed algorithm, we validate the fusion performance through both qualitative and quantitative analyses. The fusion results of the proposed method are compared with the multi-scale transform algorithms selected in Section 3 (CVT, DTCWT, LP, NSCT, NSST, GFF, and WPT) and the NestFuse[41] fusion model based on neural networks. All methods follow the parameter settings specified in the original papers. All experiments in this paper were conducted on a Windows 10 system with an Intel (R) Core (TM) i5-8300U, and all algorithms were implemented in MATLAB 2021a.

Thank you very much for your valuable comments, which have played a key role in improving the quality of our manuscript. I hope that our answers will satisfy you. If there are other questions, We hope you can give us another chance to modify them.
